# Predictors of intravenous immunoglobulin-resistant Kawasaki disease in children: a meta-analysis of 4442 cases

**DOI:** 10.1007/s00431-018-3182-2

**Published:** 2018-06-08

**Authors:** Xuan Li, Ye Chen, Yunjia Tang, Yueyue Ding, Qiuqin Xu, Lin Sun, Weiguo Qian, Guanghui Qian, Liqiang Qin, Haitao Lv

**Affiliations:** 1 Department of Cardiology, Children’s Hospital of Soochow University, Suzhou, 215003 China; 2 Institute of Pediatric Research, Children’s Hospital of Soochow University, Suzhou, China; 3 Department of Nutrition and Food Hygiene, School of Public Health, Soochow University, Suzhou, China

**Keywords:** Kawasaki disease, Intravenous immunoglobulin, Meta-analysis, Risk

## Abstract

The purpose of this study was to identify the clinical features and laboratory factors that are predictive of intravenous immunoglobulin (IVIG)-resistant Kawasaki disease. Multiple databases were searched for relevant studies on IVIG-resistant Kawasaki disease published from January 2002 to April 2017. Eligible studies were retrieved by manual review of the references. Stata 12 was used for the meta-analysis. Weighted mean differences and odds ratios with 95% confidence intervals were calculated for several indices. Twenty-eight studies involving 26,260 patients comprising 4442 IVIG-resistant Kawasaki disease patients and 21,818 IVIG-sensitive Kawasaki disease patients were included. The meta-analysis showed that the erythrocyte sedimentation rate (ESR) in the IVIG-resistant group was significantly higher than that in the IVIG-sensitive group, and that platelet count and hemoglobin levels were significantly lower in the IVIG-resistant group. The patients with oral mucosa alterations, cervical lymphadenopathy, swelling of the extremities, polymorphous rash, and initial administration of IVIG ≤ 4.0 days after the onset of symptoms were more likely to be IVIG resistant.

*Conclusion*: The initial administration of IVIG ≤ 4.0 days after the onset of symptoms increased ESR and decreased hemoglobin and platelet counts, oral mucosa alterations, cervical lymphadenopathy, swelling of the extremities, and polymorphous rash and are the risk factors for IVIG-resistant Kawasaki disease.

**Table:** 

**What is Known:** *• Recent reports on this topic are about aspartate aminotransferase (AST), alanine aminotransferase (ALT), gammaglutamyl transferase, total bilirubin, white blood cells, platelets, erythrocyte sedimentation rate (ESR), polymorphonuclear leukocytes (PMN), C-reactive protein (CRP), pro-brain natriuretic peptide (BNP), albumin, and sodium as the risk factors in the IVIG-resistant Kawasaki disease; however, no studies have been published on clinical features as predictors of IVIG resistance.*	
**What is New:** *• This meta-analysis identified the clinical features, the initial administration of IVIG ≤ 4.0 days after the onset of symptoms, and much more comprehensive laboratory indicators, such as hemoglobin, as predictors of IVIG-resistant Kawasaki disease.*

**Electronic supplementary material:**

The online version of this article (10.1007/s00431-018-3182-2) contains supplementary material, which is available to authorized users.

## Introduction

Kawasaki disease is an acute, self-limiting, systemic vascular inflammation that mainly affects the small arteries, especially the coronary arteries [39]. It is believed that during the acute period, administering large doses of immunoglobulin can reduce the risk of damage to the coronary arteries; however, 15–20% [30] of patients have intravenous immunoglobulin (IVIG)-resistant Kawasaki disease, and research [3] has shown that the probability of IVIG-resistant Kawasaki disease patients also having coronary artery lesions is nine times greater than that for IVIG-sensitive patients. Because the probability of coronary artery damage associated with IVIG-resistant Kawasaki disease is higher than that with IVIG-sensitive Kawasaki disease, if patients with IVIG-resistant Kawasaki disease can be detected and appropriately treated before additional IVIG treatments, the probability of damage to the coronary arteries would decrease, as well as the cost and hospitalization time.

There are many studies about the risk of IVIG-resistant Kawasaki disease. Japanese scholars Kobayashi et al. [20], Sano et al. [36], and Egami et al. [11] summarized the standards for the prediction of IVIG-resistant Kawasaki disease; American scholars Tremoulet et al. [42], Loomba et al. [27], and Davies et al. [7] also proposed a prediction system for the disease. At Beijing Children’s Hospital, Fu et al. [13], Yan et al. [44], and Choi et al. [6] created a scoring system based on single-center research results. Although insightful, these prediction systems lacked unity.

The aim of this study was to perform a systematic review and meta-analysis of pediatric patients reported over the past 15 years in studies published in several databases to investigate the risk factors associated with IVIG-resistant Kawasaki disease. Our results are expected to be helpful for identifying high-risk factors, providing early treatment, and reducing the occurrence of coronary artery injury in patients affected by this disease.

## Materials and methods

### Database search

Relevant multicenter or single-center studies conducted from January 2002 to April 2017 on patients with IVIG-resistant Kawasaki disease were searched. The study group was identified as those patients with IVIG-resistant Kawasaki disease, and the control group was patients with IVIG-sensitive Kawasaki disease.

Electronic databases were searched (foreign language databases, PubMed, Medline, OvidMedline, SpringerLink, China Academic Journals Full-text Database, Wanfang Data, VIP Data, and dissertation databases). The search strategy involved studies conducted from January 2002 to April 2017. Keywords used were namely “Kawasaki disease” and “IVIG resistance” or “IVIG unresponsiveness.” A manual search was conducted using reference lists of original articles. Each publication was independently reviewed and relevant information was extracted by two authors (LX and CY).

### Study selection and data extraction

The inclusion criteria were as follows: (1) diagnosed with Kawasaki disease according to Japanese diagnostic criteria and the 2017 American Heart Association common standards [14, 31] (i.e., IVIG resistance was defined as persistent or recrudescent fever (*T* ≥ 38.0 °C) at least 36 h after completion of the first IVIG infusion), (2) odds ratios (ORs) and 95% confidence intervals (CIs) provided for categorical variables in the original data and number and standard deviation provided for continuous variables, and (3) clear description of statistical methods and correct statistical analyses.

The exclusion criteria were as follows: (1) animal studies; (2) defective or poor-quality study design; (3) ORs and 95% CIs not provided for categorical variables and mean and standard deviation not provided directly or indirectly for continuous variables; and (4) review, duplicate, or unpublished literature.

The observation indices were the number of cases and control groups, days of initial administration of IVIG, hemoglobin, platelet count, erythrocyte sedimentation rate (ESR), oral mucosa, conjunctival congestion, cervical lymphadenopathy, swelling of extremities, and polymorphous rash.

A meta-analysis conducted in 2016 by Baek et al. [2] revealed that higher total bilirubin, PMN, BNP, AST, alanine transaminase (ALT), and CRP levels, and lower sodium and albumin levels are predictive of IVIG-resistant Kawasaki disease, but white blood cell count, platelet count, and ESR had no effect as predictors of IVIG-resistant Kawasaki disease. This meta-analysis showed the same results for higher total bilirubin, PMN, pro-BNP, AST, ALT, and CRP levels, and lower sodium, albumin level, and white blood cells has no effect (Supplementary 1). Considering the length of this paper, the data for these measures are not exhibited here. This paper presents the results for the clinical features and laboratory predictive factors as follows: hemoglobin, which Baek et al. [2] did not study, and platelet count and ESR, for which the results differed from those of Baek et al. [2].

### Statistical analyses

Statistical analyses were performed using Stata v. 12.0 (STATA Corp, College Station, TX, USA). Both categorical and continuous variable meta-analyses were performed. The continuous variables included platelet count, hemoglobin, and ESR. Analyses determined the relative risk of the disease for specific groups of patients (OR and 95% CI). The categorical variables included the days of initial administration of IVIG, oral mucosa alteration, conjunctival congestion, cervical lymphadenopathy, swelling of extremities, and polymorphous rash. The mean and standard deviation for each group of continuous variables were used to calculate the weighted mean difference (WMD) and 95% CI. Heterogeneity tests were performed with the use of Q and *I*^*2*^ statistics [16]. Values of *p* ≤ .10 and *I*^2^ > 50% suggested there was high statistical heterogeneity among the studies. The random-effects model was used for analysis. When *p* > .10 and *I*^2^ ≤ 50%, there was little or no statistical heterogeneity among the studies; therefore, we chose the fixed-effects model for analysis. A sensitivity analysis was conducted by omitting one study at a time to examine the influence of a single study on the overall effect sizes. Egger’s test was used to investigate publication bias [12]. If the Egger’s test revealed the *P* value of bias ≥ .1, there was no publication bias. To explore the influence of different regions on IVIG-resistant Kawasaki disease, a series of subgroup analyses were performed with meta-regression. Subgroups were selected based on different regions, such as Asian and non-Asian populations.

## Results

### Characteristics of included studies

**Fig. 1:**
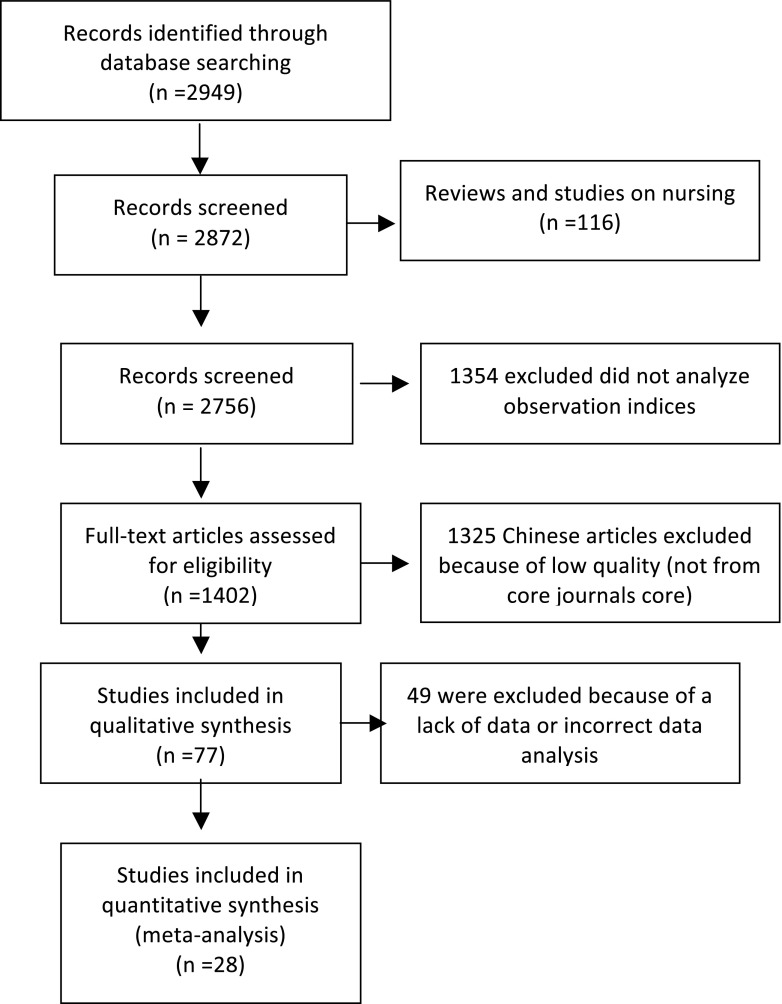
Literature selection for the meta-analysis

Our database search retrieved 2949 papers comprising 1108 in Chinese journals and 1841 in other journals. We excluded 2872 papers that were reviews, studies on nursing, or duplicate studies, as well as those that did not analyze observation indices, provide detailed data, or conform to our inclusion criteria. Among the 77 papers remaining, 49 were excluded because of a lack of data or incorrect data analysis. Finally, 28 papers with 26,260 cases were selected, with 4442 cases in the IVIG-resistant group and 21,818 in the IVIG-sensitive group (Fig. 1). The studies were conducted in Japan (*n* = 7), China (*n* = 10, including four articles from Chinese Taipei, North America (*n* = 5)), and Korea (*n* = 6). The general characteristics of the groups from the selected literature are shown in Table 1.

**Table 1:** Characteristics of patients included in the study

Author	Year	Mean age (months)	Sex (female/male)	Number of patients of IVIG-resistant, *n*	Number of patients of IVIG-sensitive, *n*	The rate of IVIG-resistant(%)	Location
Durongpisitkul et al. [10]	2003	Average 26.35	52/68	14	106	11.67%	China
Kobayashi et al. [20]	2006	29.1 ± 22.1	231/315	112	434	20.51%	Japan
Sano et al. [36]	2007	Average 27.24	59/53	22	90	19.64%	Japan
Egami et al. [11]	2006	Average 27.6	136/184	41	279	12.81%	Japan
Muta et al. [28]	2006	Average 30.61	4822/6544	1855	9511	16.32%	Japan
Du et al. [9]	2006	31.2 ± 26.4	370/682	135	917	12.83%	China
Cha et al. [4]	2008	Average 35.37	17/34	18	33	35.29%	Korea
Uehara et al. [43]	2008	–	2643/3687	1286	5044	20.32	Japan
Ashouri et al. [1]	2008	35.13	83/113	40	156	20.41	North America
Tremoulet et al. [42]	2008	–	–	60	302	16.57	North America
Piao et al. [33]	2009	25.48	75/147	37	185	16.67	China
Rigante et al. [35]	2010	23.8	12/20	5	27	15.63	North America
Kuo et al. [22]	2010	19.25	39/92	20	111	15.27	China
Hwang et al. [17]	2011	28.77	103/126	23	206	10.04	Korea
Sleeper et al. [38]	2011	39.22	74/124	27	171	13.64	North America
Liu et al. [26]	2012	–	110/268	24	354	6.35	China
Yan et al. [44]	2012	29.92	77/142	21	198	9.59	China
Sato et al. [37]	2013	26.7	43/62	21	84	20.00	Japan
Fu et al. [13]	2013	–	431/746	211	966	17.93	China
Kim et al. [18]	2013	29.24	51/84	22	113	16.30	North America
Ou-Yang et al. [32]	2013	19.86	23/40	5	58	7.94	China
Choi et al. [6]	2014	33.47	231/342	158	415	27.57	Korea
Lee et al. [24]	2014	38.22	44/47	11	80	12.09	Korea
Lin et al. [25]	2016	22.8	74/107	22	159	12.15	China
Nakagama et al. [29]	2016	33.75	62/109	54	117	31.58	Japan
Kim et al. [19]	2016	31.9	302/401	118	585	16.79	Korea
Lee et al. [23]	2016	–	128/159	34	253	11.85	Korea
Tang et al. [41]	2016	–	584/326	46	864	5.05	China

### Indices of high-risk factors

**Fig. 2:**
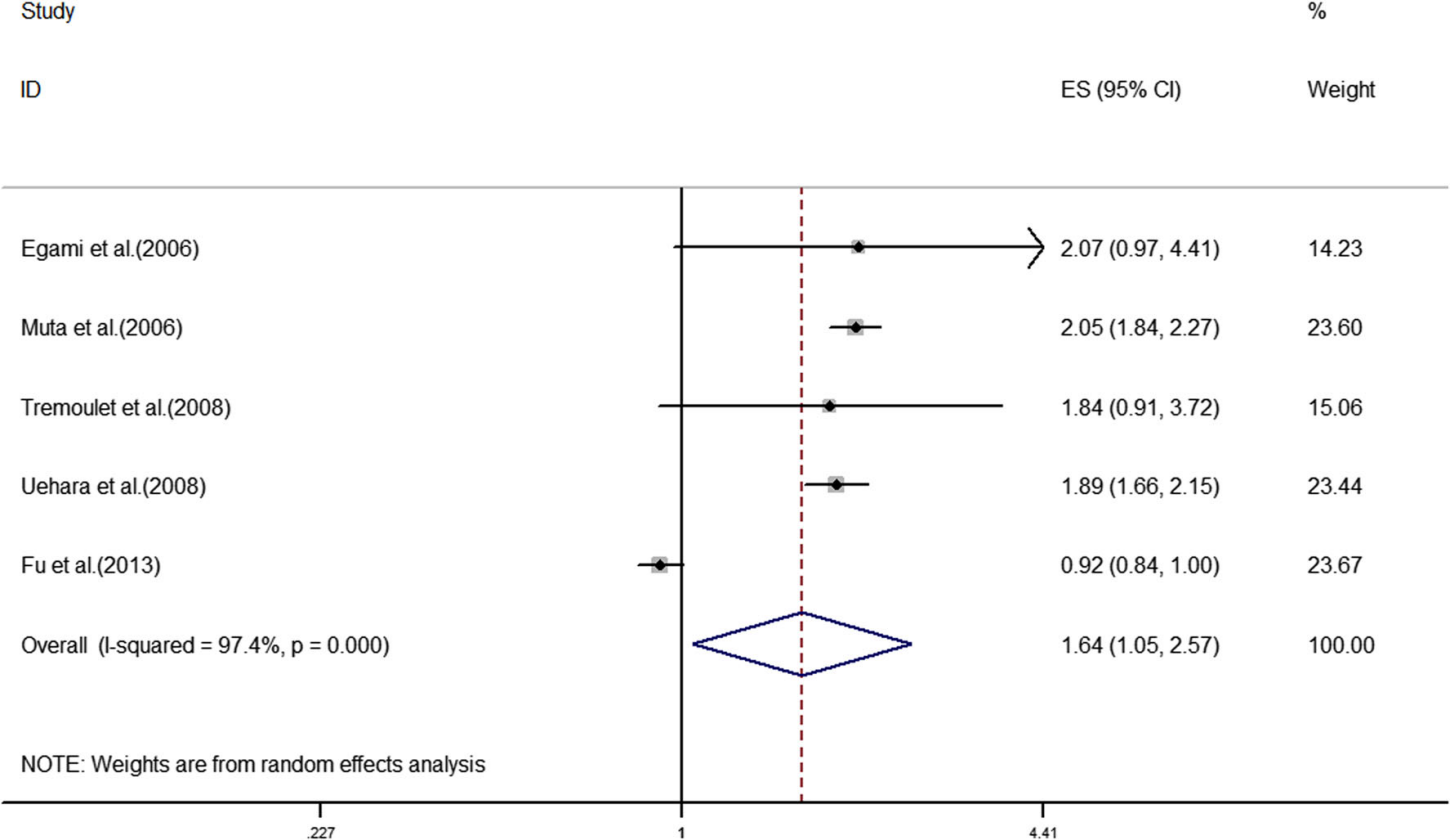
Prevalence of intravenous immunoglobulin (IVIG)-resistant Kawasaki disease among patients who received IVIG treatment ≤ 4.0 days after the onset of symptoms

**Fig. 3:**
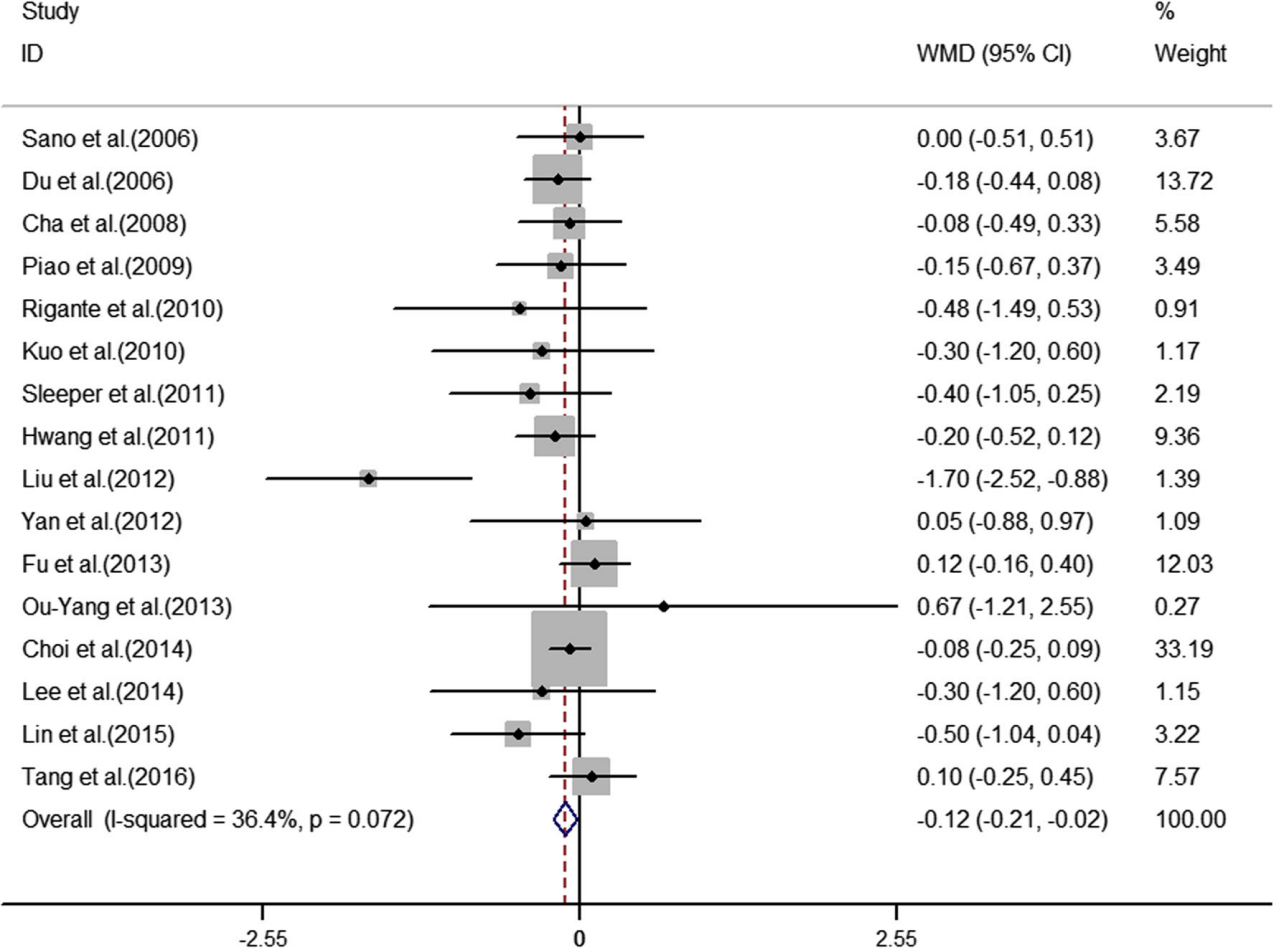
Hemoglobin as a predictive index for resistance to intravenous immunoglobulin therapy in Kawasaki disease

**Fig. 4:**
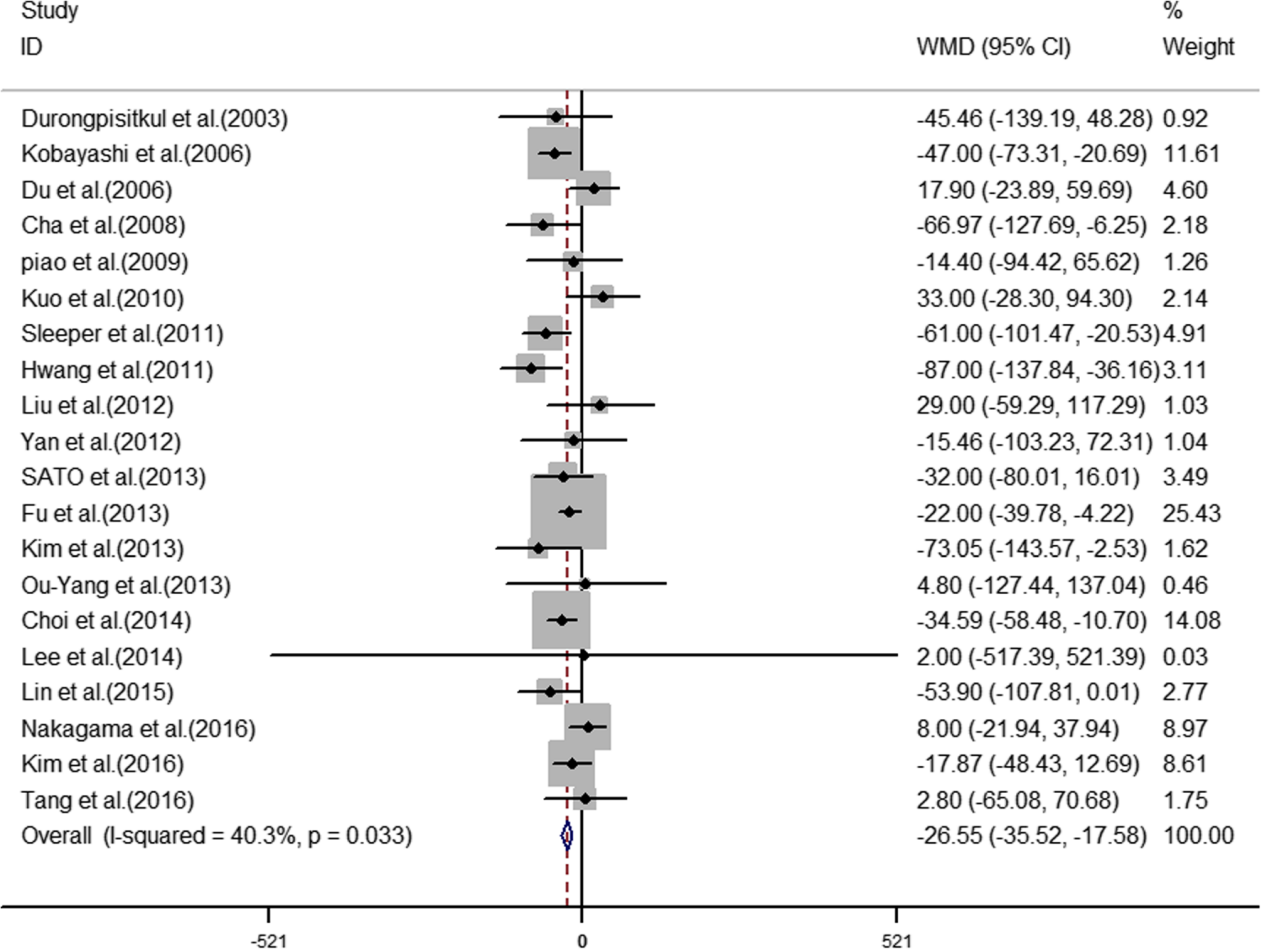
Platelet count as a predictive index for intravenous immunoglobulin resistance in Kawasaki disease

**Fig. 5:**
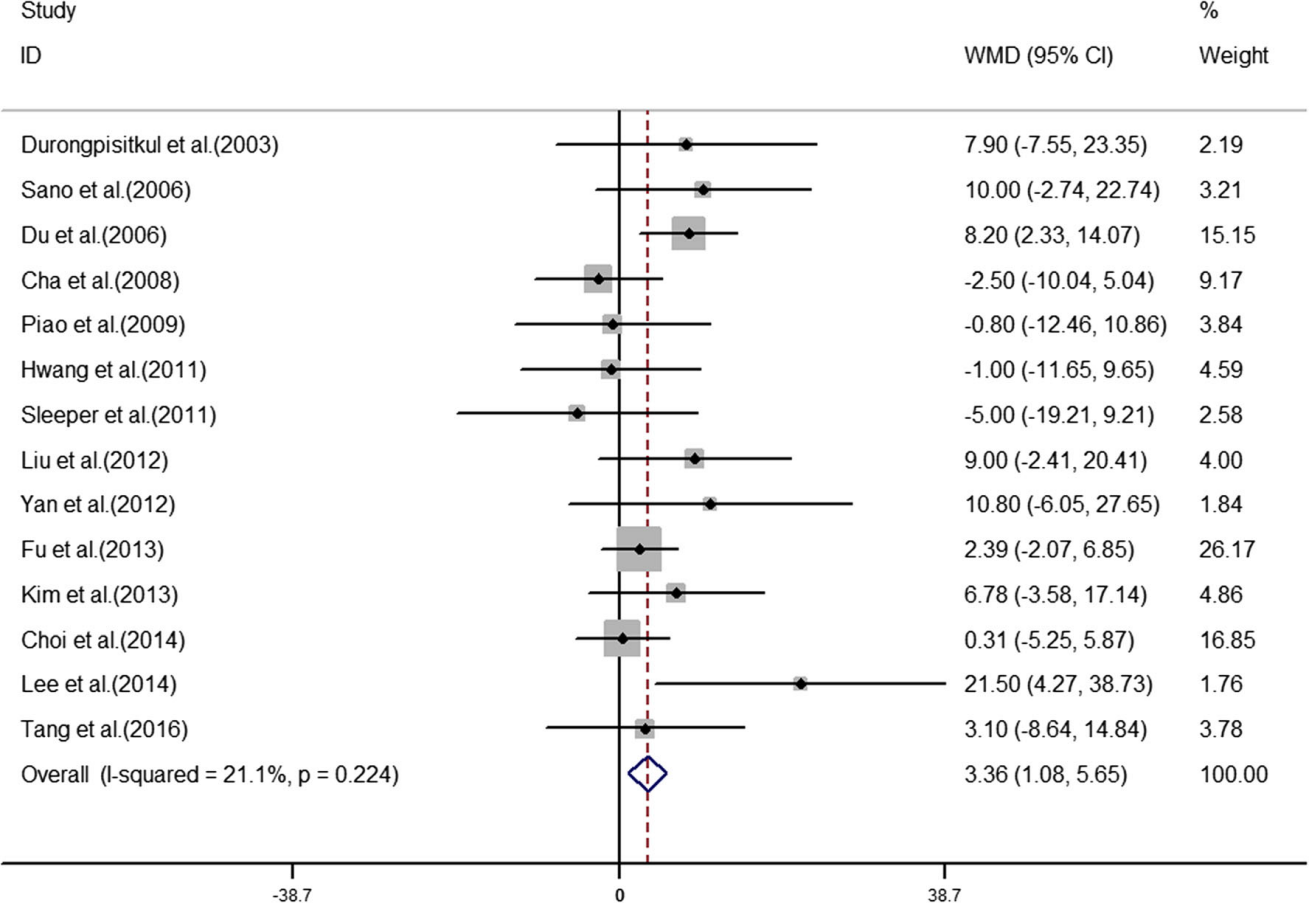
Erythrocyte sedimentation rate (ESR) as a predictive index for intravenous immunoglobulin resistance in Kawasaki disease

**Fig. 6:**
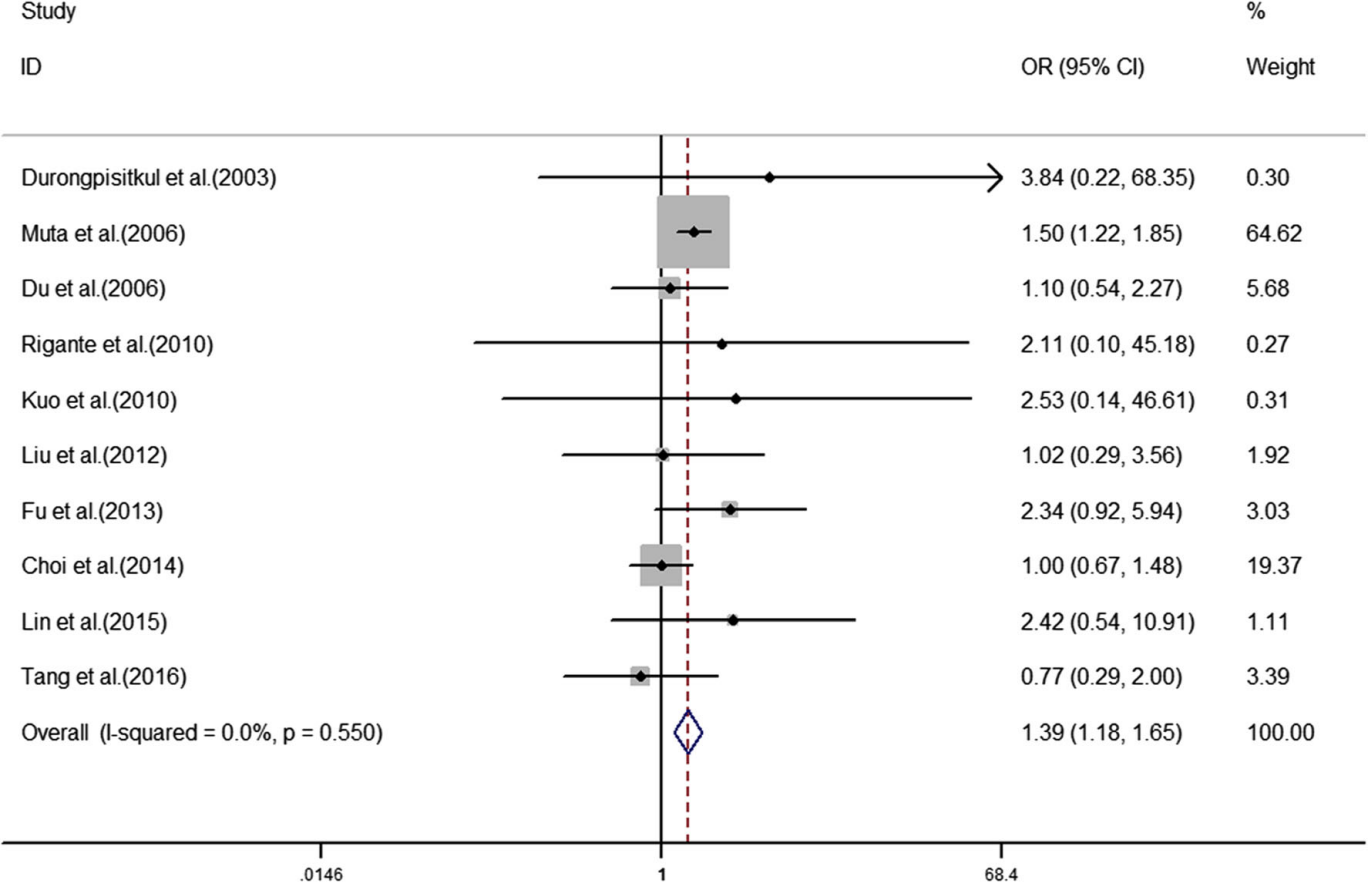
Changes in oral mucosa as a predictive index for intravenous immunoglobulin resistance in Kawasaki disease

**Fig. 7:**
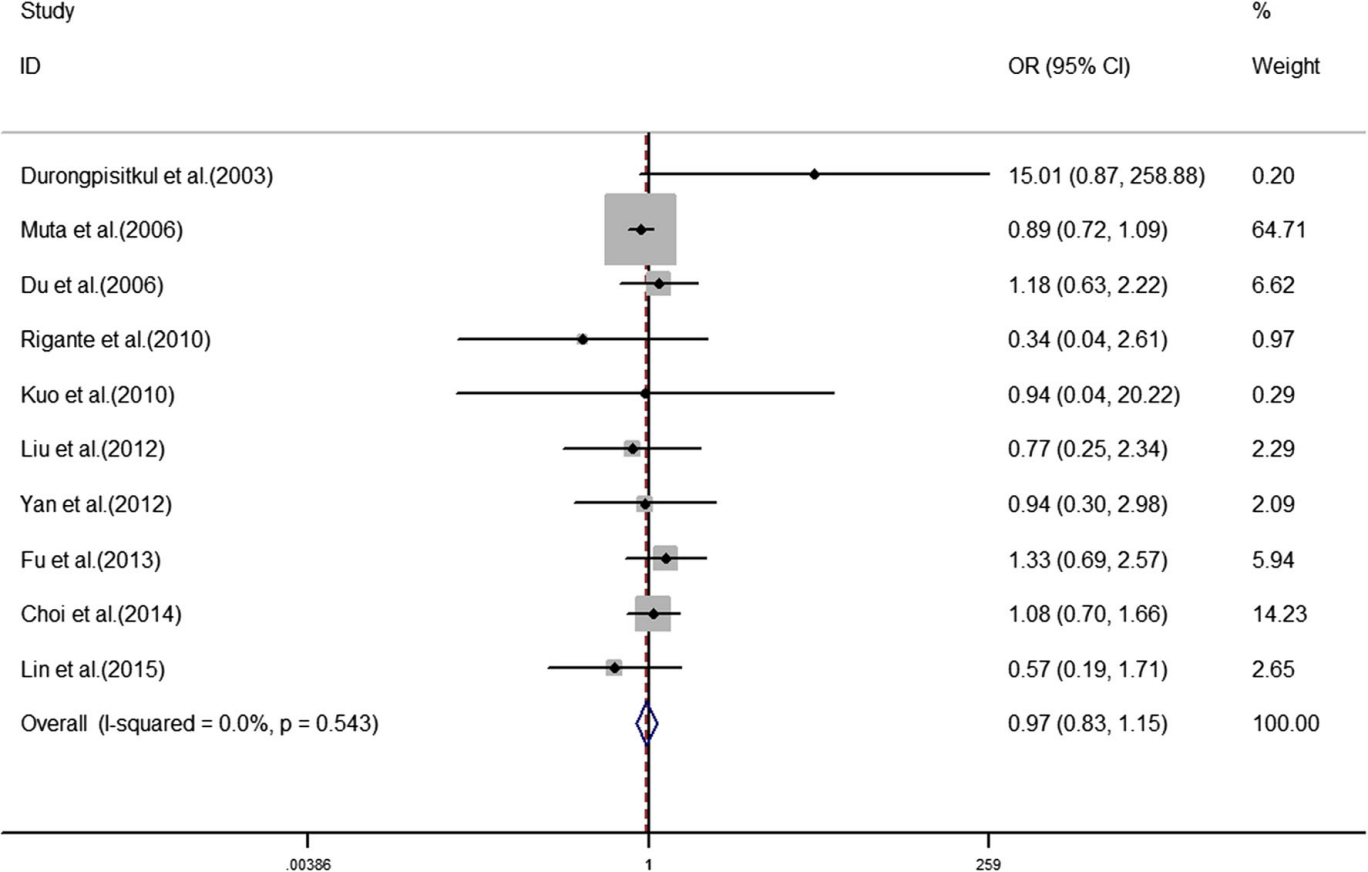
Conjunctival congestion as a predictive index for intravenous immunoglobulin resistance in Kawasaki disease

**Fig. 8:**
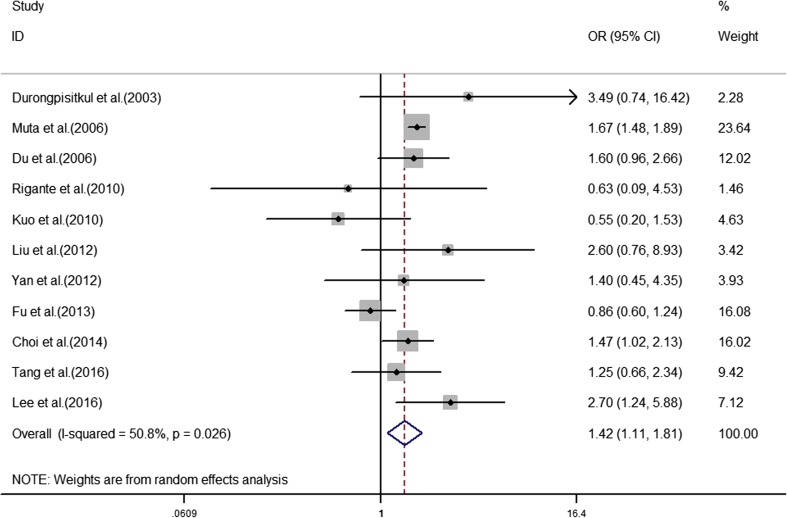
Cervical lymphadenopathy as a predictive index for intravenous immunoglobulin resistance in Kawasaki disease

**Fig. 9:**
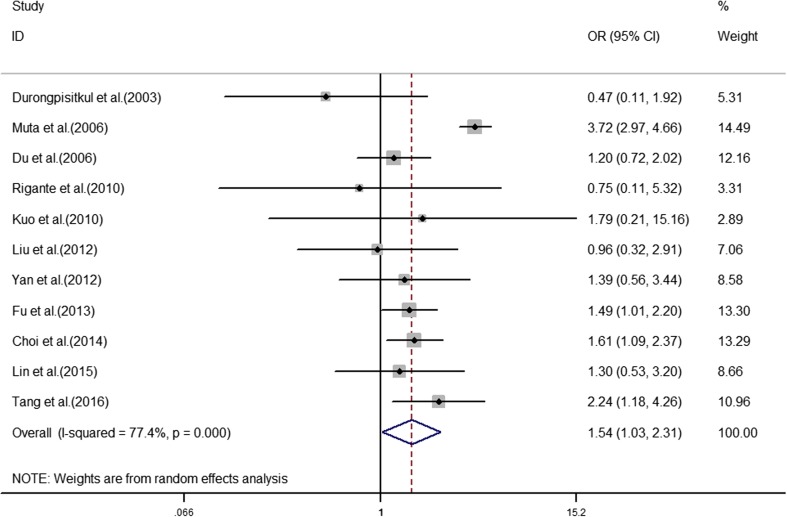
Swelling of the extremities as a predictive index for intravenous immunoglobulin resistance in Kawasaki disease

**Fig. 10:**
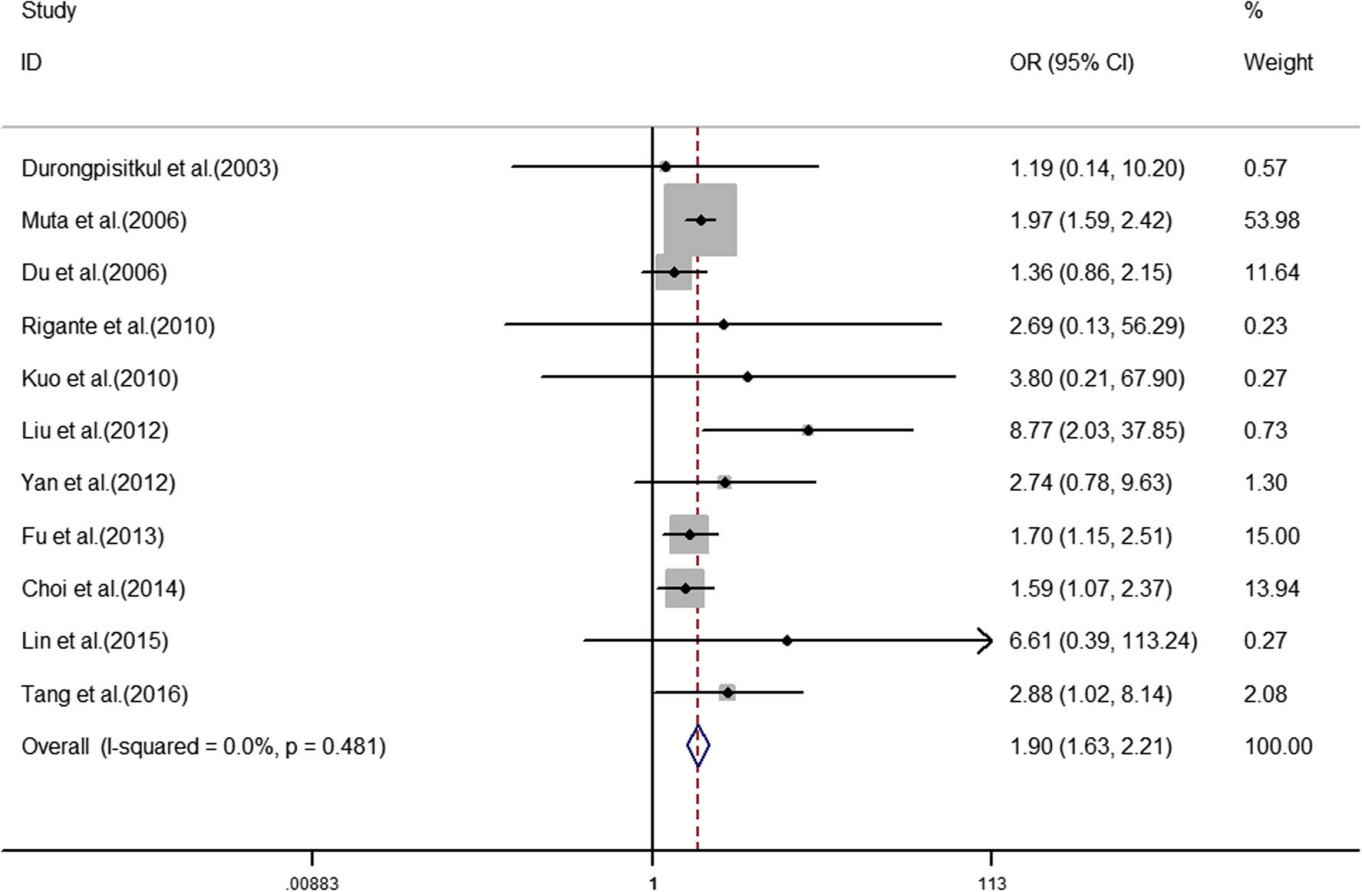
Polymorphous rash as a predictive index for intravenous immunoglobulin resistance in Kawasaki disease

The analyses of the high-risk factors are shown in Table 2. Treatment time ≤ 4.0 days was more likely to not result in IVIG-sensitive Kawasaki disease, with an OR value of 1.64 and 95% CI of 1.05–2.57 (Fig. 2). IVIG-resistant patients had a significantly lower hemoglobin value than IVIG-sensitive patients (Fig. 3), with WMD = − 26.55 and 95% CI = − 35.52, − 17.58. IVIG-resistant patients had significantly lower platelet counts than IVIG-sensitive patients (Fig. 4), with WMD = − 26.55 and 95% CI = − 35.52, − 17.58. IVIG-resistant patients had significantly higher ESR values than IVIG-sensitive patients (Fig. 5), with WMD = 3.36 and 95% CI = 1.08–5.65. IVIG-resistant patients were more likely to have changes in oral mucosa than IVIG-sensitive patients (Fig. 6), with OR = 1.39 and 95% CI = 1.18–1.65. The difference in conjunctival congestion in each group was not statistically significant (Fig. 7), with OR = 0.97 and 95% CI = 0.83–1.15. IVIG-resistant patients were more likely to have cervical lymphadenopathy than IVIG-sensitive patients (Fig. 8), with OR = 1.42 and 95% CI = 1.11–1.81. IVIG-resistant patients were more likely to have swelling of the extremities than IVIG-sensitive patients (Fig. 9), with OR = 1.54 and 95% CI = 1.03–2.31. Patients with polymorphous rash were significantly more likely not to be sensitive to IVIG (Fig. 10), with OR = 1.90, and 95% CI = 1.63–2.21.

**Table 2:** Pooled estimates of indices of high-risk factors on intravenous immunoglobulin (IVIG) resistance

Variables	Number of trials	I2 (%)	Net change (95%CI)	*p*
Initiation of IVIG treatment	5	97.4	1.64 (1.05, 2.57)	0.03
Hemoglobin	16	36.4	− 0.12 (− 0.21, − 0.02)	0.02
Platelet count	20	40.3	− 26.55 (− 35.52, − 17.58)	< 0.001
Erythrocyte sedimentation rate	14	21.1	3.36 (1.08, 5.65)	0.004
Changes in oral mucosa	10	0	1.39 (1.18, 1.65)	< 0.001
Conjunctival congestion	10	0	0.97 (0.83, 1.15)	0.755
Cervical lymphadenopathy	11	50.8	1.42 (1.11, 1.81)	0.06
Swelling of extremities	11	77.4	1.54 (1.03, 2.31)	< 0.001
Polymorphous rash	11	0	1.90 (1.63, 2.21)	< 0.001

### Sensitivity analyses and publication bias

Omitting one study at a time for the platelet count data produced WMD values from − 29.956 (95% CI − 39.354, −20.557) to − 23.864 (95% CI − 33.402, − 14.327). Omitting one study at a time for the erythrocyte sedimentation rate data gave WHD values from 2.498 (95% CI 0.019–4.977) to 3.981 (95% CI 1.477–6.485). Omitting one study at a time for cervical lymphadenopathy data showed OR values from 1.309 (95% CI 1.078–1.591) to 1.640 (95% CI 1.473–1.825). Omitting one study at a time for swelling of the extremities data gave OR values from 1.461 (95% CI 1.189–1.795) to 1.657 (95% CI 1.111–2.471). Omitting one study at a time for polymorphous rash data showed OR values from 1.814 (95% CI 1.455–2.261) to 1.967 (95% CI 1.674–2.310). Egger’s tests for hemoglobin and platelet count showed no publication bias (*p* = .18 and *p* = .97, respectively).

### Subgroup analyses

The results of our subgroup analyses are shown in Table 3. Subgroups were selected based on different regions, such as Asian and non-Asian. Hemoglobin, platelet count, ESR, oral mucosa, conjunctival congestion, cervical lymphadenopathy, swelling of extremities, and polymorphous rash were analyzed. For the initiation of IVIG treatment, we had only five studies; therefore, we did not conduct a subgroup analysis for this variable. The summary WMDs for hemoglobin were 0.14 (95% CI − 0.29, 0.01) and − 0.42(95% CI − 0.97, 0.13) for the studies in Asian and non-Asian populations, respectively, without any significant region-specific differences (P_difference_ = 0.07). The summary WMDs for platelet count were − 23.22 (95% CI − 36.88, − 9.55) and − 63.99 (95% CI − 40.57, − 13.58) for the studies in Asian and non-Asian populations, respectively, indicating there was a significant region-specific difference (P_difference_ = 0.03). Asian patients with high ESR are more likely to be IVIG resistant, but there was no significant difference between IVIG-sensitive and IVIG-resistant patients in non-Asian patients. Asian patients with changes in oral mucosa, cervical lymphadenopathy, swelling of the extremities, and polymorphous rash were more likely to be IVIG resistant, but there was no significant difference between IVIG-sensitive and IVIG-resistant patients in non-Asian patients. For conjunctival congestion, there was no difference in Asian and non-Asian patients between those who were IVIG-sensitive and those who were IVIG resistant.

**Table 3:** Subgroup analyses for meta-analysis of the risk of intravenous immunoglobulin (IVIG)-resistant Kawasaki disease

Factor	Geographic area	Number of studies	WMD (95% CI)	P_heterogeneity_	*I*^2^(%)	P_difference_
Hemoglobin	Asian [4, 6, 9, 13, 17, 22, 24–26, 32, 33, 36, 41, 44]	14	− 0.14 (− 0.29, 0.01)	0.05	41.8	0.07
	Non-Asian [35, 38]	2	− 0.42 (− 0.97, 0.13)	0.90	0	
Platelet count	Asian [4, 6, 9, 10, 13, 17, 19, 20, 22, 24–26, 29, 32, 33, 37, 41, 44]	18	− 23.22 (− 36.88, − 9.55)	0.06	37.1	0.03
	Non-Asian [18, 38]	2	− 63.99 (−40.57, − 13.58)	0.77	0	
Erythrocyte sedimentation rate	Asian [4, 6, 9, 10, 13, 17, 18, 24, 26, 33, 36, 41, 44]	12	3.76 (0.76, 6.77)	0.20	25.3	0.22
	Non-Asian [18, 38]	2	1.94 (− 9.43, 13.30)	0.19	42.0	
Changes in oral mucosa	Asian [6, 9, 10, 13, 22, 25, 26, 28, 41]	9	1.37 (1.16, 1.62)	0.46	0	0.55
	Non-Asian [35]	1	2.11 (0.10, 45.18)	NA	NA	
Conjunctival congestion	Asian [6, 9, 10, 13, 22, 25, 26, 28, 41, 44]	9	0.98 (0.83, 1.16)	0.54	0	0.54
	Non-Asian [35]	1	0.34 (0.83, 1.15)	NA	NA	
Cervical lymphadenopathy	Asian [6, 9, 10, 13, 22, 23, 26, 28, 41, 44]	10	1.43 (1.12,1.84)	0.02	53.9	0.03
	Non-Asian [35]	1	.63 (0.09, 4.53)	NA	NA	
Swelling of extremities	Asian [6, 9, 10, 13, 22, 23, 25, 26, 28, 41, 44]	10	1.58 (1.05, 2.38)	0.00	79.1	0.00
	Non-Asian [35]	1	0.75 (0.11, 5.32)	NA	NA	
Polymorphous rash	Asian [6, 9, 10, 13, 22, 23, 25, 26, 28, 41, 44]	10	1.84 (1.55, 2.18)	0.39	5.2	0.48
	Non-Asian [35]	1	2.69 (0.13, 56.30)	NA	NA	

*WMD* weighted mean difference, *95%CI* 95% confidence intervals

Our results on platelet count and ESR differed from those of Baek et al. [2]. We included more studies, for a longer study period, with subgroup analyses (Table 4). The results revealed that the difference in platelet count among Chinese, Korean, Japanese, and non-Asian patients was significant, and the difference in erythrocyte sedimentation rate was statistically significant in Chinese patients, but not in Korean, Japanese, and non-Asian patients.

**Table 4:** Subgroup analyses for meta-analysis of platelet count and erythrocyte sedimentation rate

Factor	Geographic area	Number of studies	WMD (95% CI)	P_heterogeneity_	*I*^2^(%)	P_difference_
Platelet count	Japan	2	− 43.535 (−66.61, − 20.46)	0.59	0	.03
	China	10	− 14.221 (−28.32, − 0.13)	0.43	0.4	
	Korea	6	− 32.851 (−60.42, − 5.29)	0.02	61.6	
	Non-Asian	2	− 63.985 (95% CI − 99.09, − 28.88)	0.77	0	
Erythrocyte sedimentation rate	Japan	1	10.000 (− 2.74, 22.74)	–	–	.22
	China	6	4.588 (1.50, 7.67)	0.50	42.0	
	Korea	5	2.162 (− 3.75, 8.08)	0.12	44.7	
	Non-Asian	2	1.936 (− 9.43, 13.30)	0.190	21.1	

## Discussion

This meta-analysis showed that differences in the timing of initiation of IVIG treatment (≤ 4.0 days), hemoglobin level, platelet count, ESR, oral mucosa features, cervical lymphadenopathy, swelling of the extremities, and polymorphous rash between IVIG-resistant and IVIG-sensitive patients were statistically significant.

### Initial administration of IVIG

Our study found that initial administration of IVIG ≤ 4.0 days rather than > 4.0 days after the onset of symptoms resulted in Kawasaki disease that was more likely to be IVIG resistant (*p* = .03). Among the included studies, Fu et al. [13], Egami et al. [11], and Tremoulet et al. [42] concluded that initial administration of IVIG ≤ 4.0 days after the onset of symptoms might not correlate with IVIG resistance; whereas, two other studies showed a relationship, and the overall combined effect revealed the relationship between the initial administration of IVIG and IVIG resistance. The symptoms of Kawasaki disease always appear after fever; therefore, if the patient has confirmed Kawasaki disease for ≤ 4.0 days, it suggests the severity of the disease, which is perhaps why the patients treated with IVIG ≤ 4.0 days were more susceptible to IVIG resistance. In the acute phase of Kawasaki disease, the inflammatory reaction continues, and the early use of IVIG cannot block the inflammatory mediators that continue to be released. If IVIG is used within 4 days, the inflammatory reaction will continue; therefore, the possibility of continuous fever might be likely.

### Hemoglobin and ESR

Straface et al. [40] found that the inflammatory reaction in patients with Kawasaki disease changes in the serum redox state, with increased expression of inducible nitric oxide synthase in monocytes and neutrophils. It has been suggested that this pro-oxidant status of the blood could also alter the homeostasis of red blood cells (RBCs), resulting in a type of premature aging in these circulating cells that could lead to anemia and the formation of blood clots. Decreased glycophorin A and CD47 expression, as well as the externalization of phosphatidylserine, were measured in RBCs from patients with Kawasaki disease during the early phase of the disease. The number of RBCs, hemoglobin values, mean corpuscular volume, and hematocrit were significantly decreased in these patients. Alterations in RBC structure and function might independently and synergistically impair blood flow and induce vascular occlusion, whereas premature aging of RBCs and their consequent removal from circulation might be a risk factor for anemia. RBC aging, inflammation, and thrombosis result in increased ESR. In this study, the IVIG-resistant patients had a significantly lower hemoglobin level and significantly higher ESR than IVIG-sensitive patients; however, the differences were not significant among each subgroup. The results for hemoglobin in each subgroup showed no relationship to IVIG resistance; however, a relationship was observed in the combined results. One Korean study [24] and one Chinese study [9] showed a strong relationship between ESR and Kawasaki disease; the other 12 studies showed a weak relationship.

### Platelet count

Del et al. [8] suggested that the formation of heterotypic platelet–leucocyte aggregates, which is dependent on platelet activation, and leucocyte–RBC–platelet aggregates could at least partially be associated with the release of pro-aggregating factors (e.g., arachidonate) and/or with changes in the expression of molecules on the cell surface, including P-selectin. This crosstalk between activated platelets and leucocytes operates through several systems, including the interaction of P-selectin with P-selectin glycoprotein ligand-1 (PSGL-1). P-selectin and PSGL-1 are vascular adhesion molecules that play an important role in the inflammatory response by mediating the interaction of leucocytes, which stimulates endothelium and platelets bound within the vicinity of vascular injury. P-selectin captures leucocytes from the blood to bring them into contact with the endothelial cell surface on the blood vessel wall where P-selectin–PSGL-1 interaction supports leucocyte rolling, platelet activation, and aggregation, which leads to a cascade of reactions that promote inflammation and thrombosis; therefore, we can conclude that the platelet count is associated with the inflammatory reaction of Kawasaki disease and can speculate that the number of platelets is positively correlated with the severity of inflammation.

This study also confirmed that platelet count can predict IVIG-resistant Kawasaki disease. The results of 20 studies were combined in this study comprising 18 Asian studies [4, 6, 9, 10, 13, 17, 19, 20, 22, 24–26, 29, 32, 33, 37, 41, 44] and 2 North American studies [18, 38]. Each subgroup analysis showed the results had statistical significance; however, Baek et al. [2] reported that ESR and platelet count could not predict IVIG-resistant Kawasaki disease from their statistical meta-analysis, which is contrary to the results of the present study. Baek et al. [2] included 7 papers reporting ESR and 10 papers reporting platelet count, where we included 14 papers reporting ESR and 20 papers reporting platelets, giving the present study a larger sample size with higher reliability. In addition, the subgroup analyses of platelet count showed that the differences in platelet count in Chinese, Korean, Japanese, and non-Asian patients were significant. For erythrocyte sedimentation rate, the difference was significant in Chinese, but not in Korean, Japanese, and non-Asian patients. The results differed by ethnicity.

### Clinical features

IVIG-resistant Kawasaki disease with fever over a long period can have different clinical features. Ram et al. [34] found that prolonged fever, wider dispersion of symptoms, and pyuria were significantly associated with the development of coronary lesions, all of which the Kawasaki disease patients had. Choi et al. [6] found that cervical lymphadenopathy is a risk factor for IVIG resistance, and Fu et al. [13] believed that polymorphous rash and perianal change are risk factors for IVIG resistance. Yan et al. [44] suggested that clinical features cannot predict IVIG-resistant Kawasaki disease; however, this study found that changes in oral mucosa, cervical lymphadenopathy, swelling of extremities, and polymorphous rash can predict IVIG-resistant Kawasaki disease, whereas conjunctival congestion cannot. Hartas et al. [15] showed that patients with Kawasaki disease who also have acute arthritis are at high risk for being IVIG-resistant, but because of the lack of relevant studies, we did not include this factor in our meta-analysis.

In subgroup analyses, Asian patients with changes in oral mucosa, cervical lymphadenopathy, swelling of the extremities, and polymorphous rash were more likely to be IVIG resistant, but in non-Asian patients, there was no significant difference among these symptoms and IVIG resistance. For conjunctival congestion, neither Asian nor non-Asian patients exhibited any difference between IVIG sensitivity and IVIG resistance.

This study was aimed to explore the risk factors associated with IVIG-resistant Kawasaki disease through studying clinical features and laboratory index, which would provide evidences for treatment regimens in these patients. Chen et al. [5] and Yang et al. [45] conducted the meta-analysis, which pointed out that the early application of intravenous immunoglobulin plus corticosteroid can reduce the incidence of coronary artery abnormalities. A prospective study was conducted by Kobayashi et al. [21] and after putting forward the Kobayashi score, they found that among IVIG-resistant high-risk patients (Kobayashi score, 5 or higher), the incidences of treatment failure and coronary artery abnormalities were more frequent in the IVIG group than in the IVIG + PSL group. The clinical and coronary outcomes were similar among low-risk patients (Kobayashi score 0–4). A prediction model to select the appropriate treatment and alleviate complications in IVIG-resistant Kawasaki disease was warranted in the future.

## Study limitations

There were several limitations to our study. First, most studies used were retrospective and few multicenter studies were included. Second, because of language constraints, few Japanese articles were included. Third, because the original articles did not provide the data on age-adjusted z-scores of hemoglobin, we had no statistic on zHgb.

## Conclusion

The risk factors for IVIG-resistant Kawasaki disease are the initial administration of IVIG ≤ 4.0 days after the onset of symptoms, increased ESR, decreased hemoglobin and platelet count, changes in oral mucosa, cervical lymphadenopathy, swelling of extremities, and polymorphous rash.

## Electronic supplementary material

ESM 1 (DOCX 4290 kb)

ESM 2 (DOC 67 kb)

## Abbreviations

BNP: Brain natriuretic peptide
ALT: Alanine transaminase
AST: Aspartate aminotransferase
CI: Confidence interval
CRP: C-reactive protein
IVIG: Intravenous immunoglobulin
ESR: Erythrocyte sedimentation rate
OR: Odds ratio
PMN: Polymorphonuclear leukocytes
WMD: Weighted mean difference
