# Supplementary material for: Predictors of intravenous immunoglobulin-resistant Kawasaki disease in children: a meta-analysis of 4442 cases
Source: Eur J Pediatr. 2018 Jun 8;177(8):1279–92. doi: 10.1007/s00431-018-3182-2 (PMC6061038; doi:10.1007/s00431-018-3182-2)
Supplement: Supplementary file 1 — (DOCX 4290 kb) [file 431_2018_3182_MOESM1_ESM.docx]

**
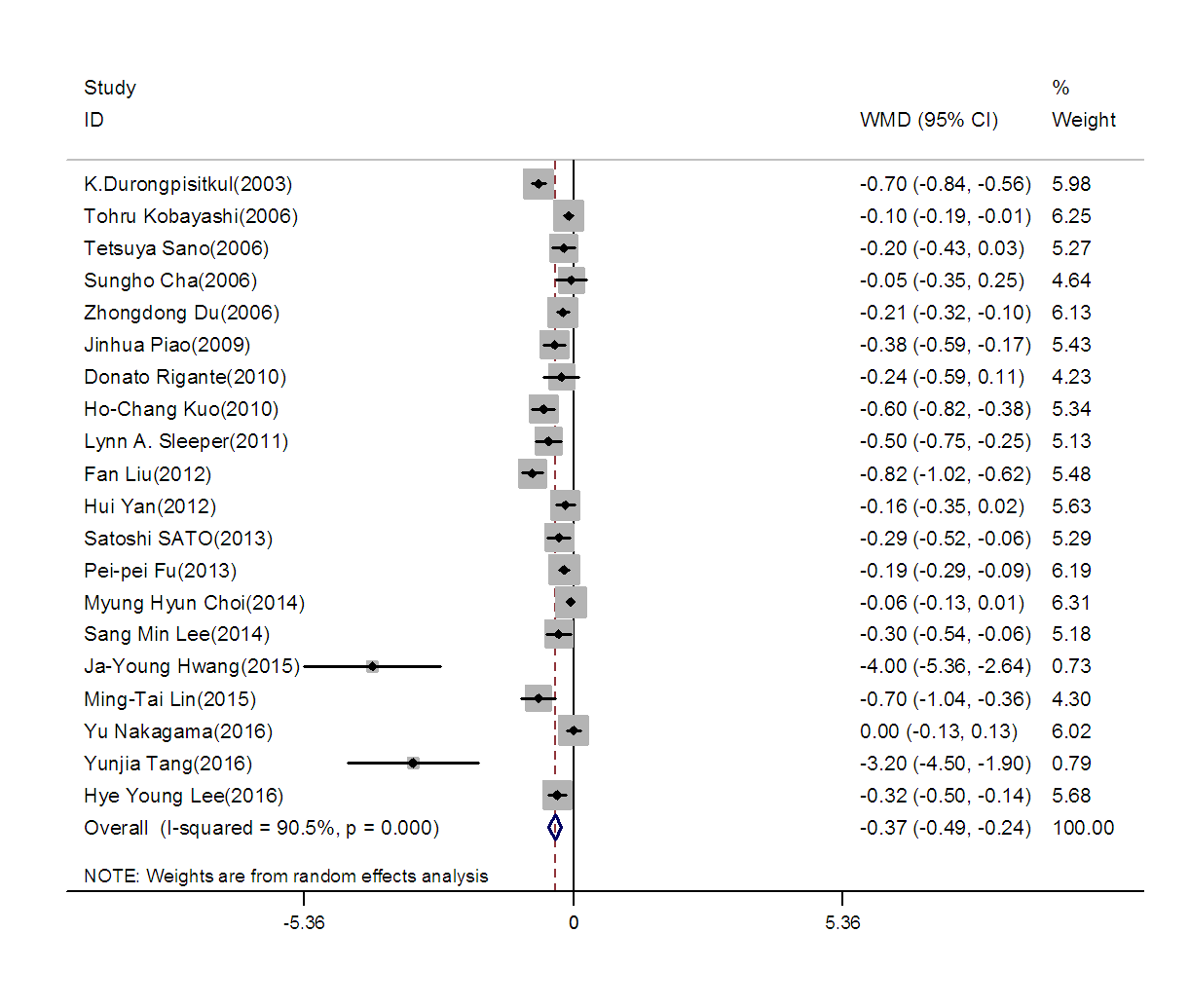
Fig. 11 Albumin as a predictive index for resistance to intravenous immunoglobulin therapy in Kawasaki disease.**

**
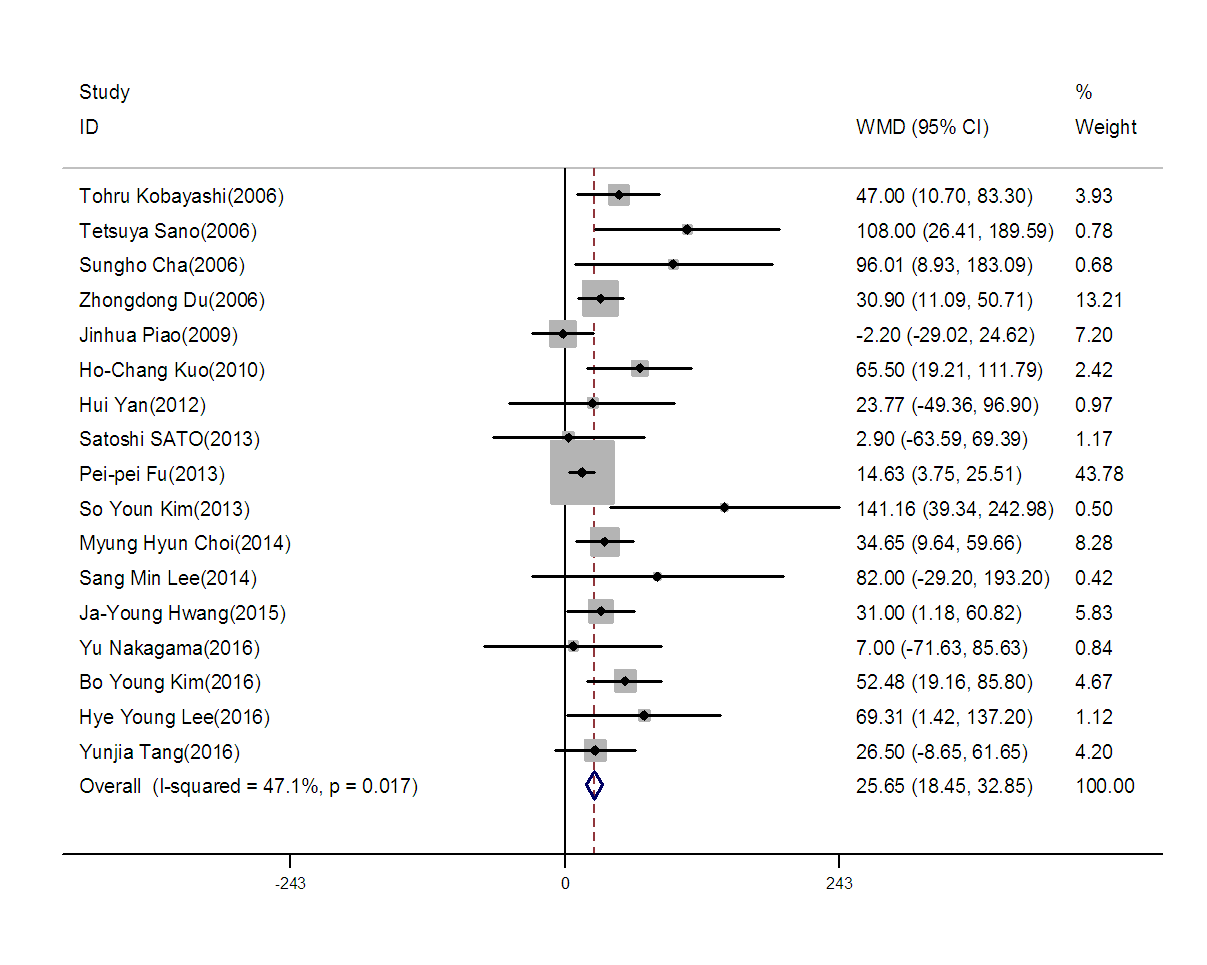
Fig. 12 ALT as a predictive index for resistance to intravenous immunoglobulin therapy in Kawasaki disease**.


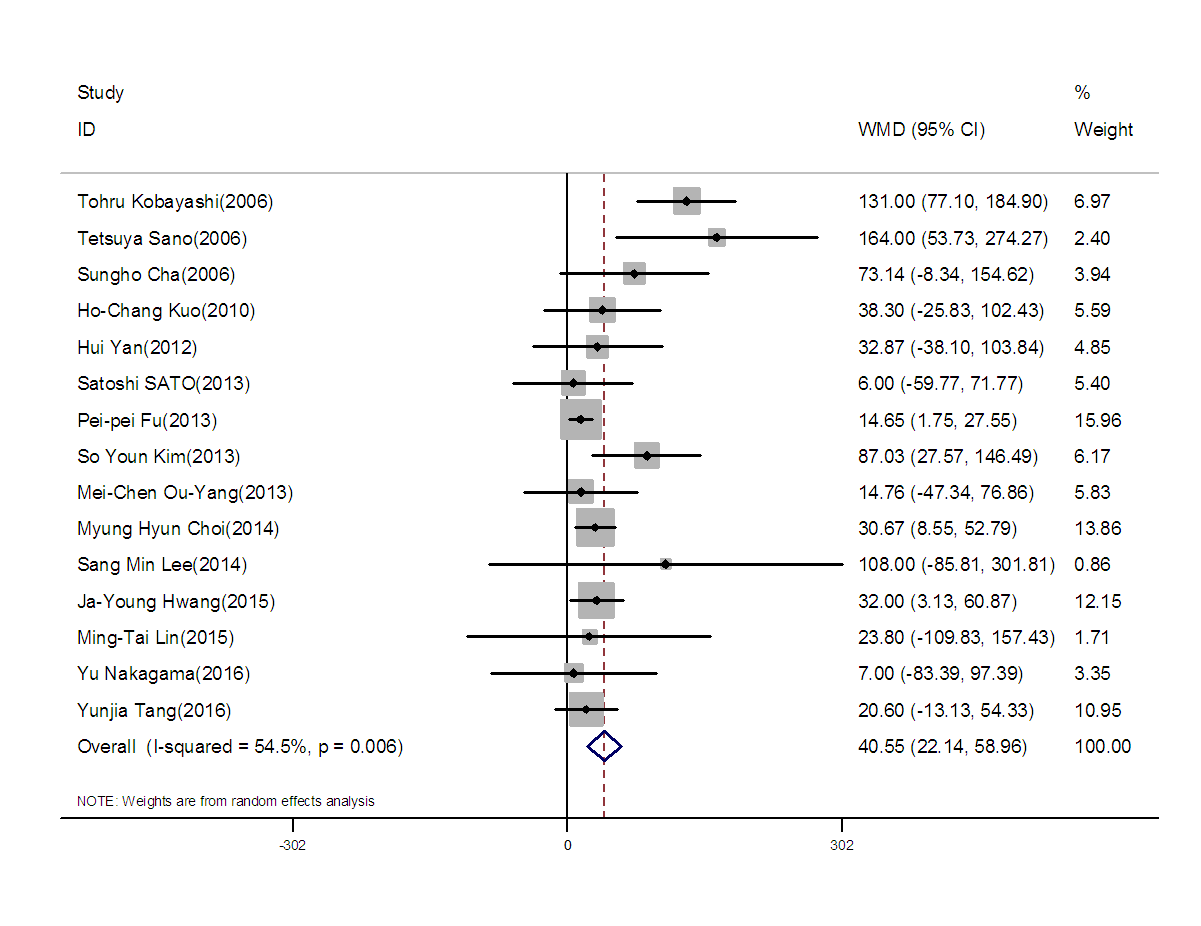


**Fig. 13 AST as a predictive index for intravenous immunoglobulin resistance in Kawasaki disease.**

**
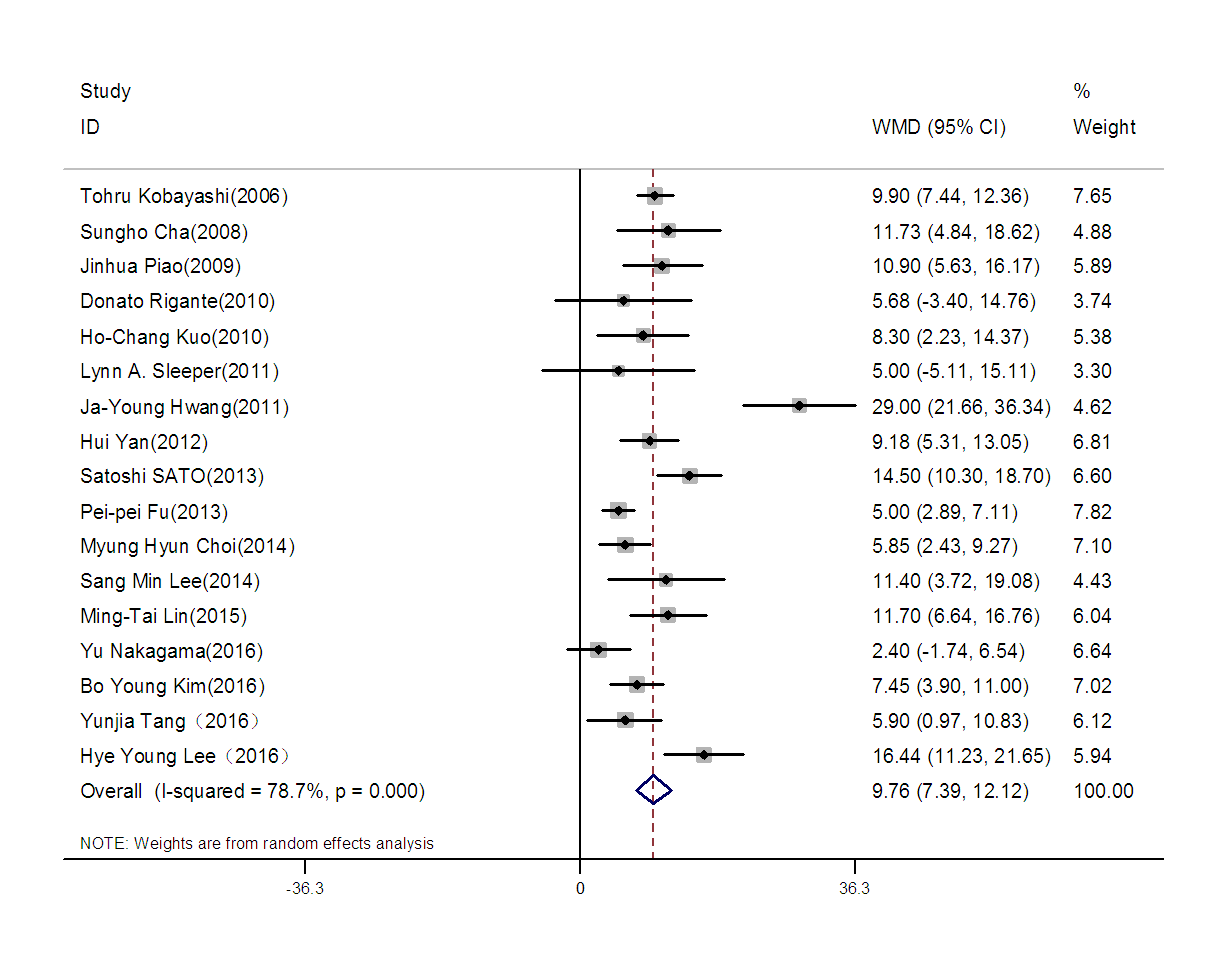
Fig. 14 PMN as a predictive index for intravenous immunoglobulin resistance in Kawasaki disease.**

**
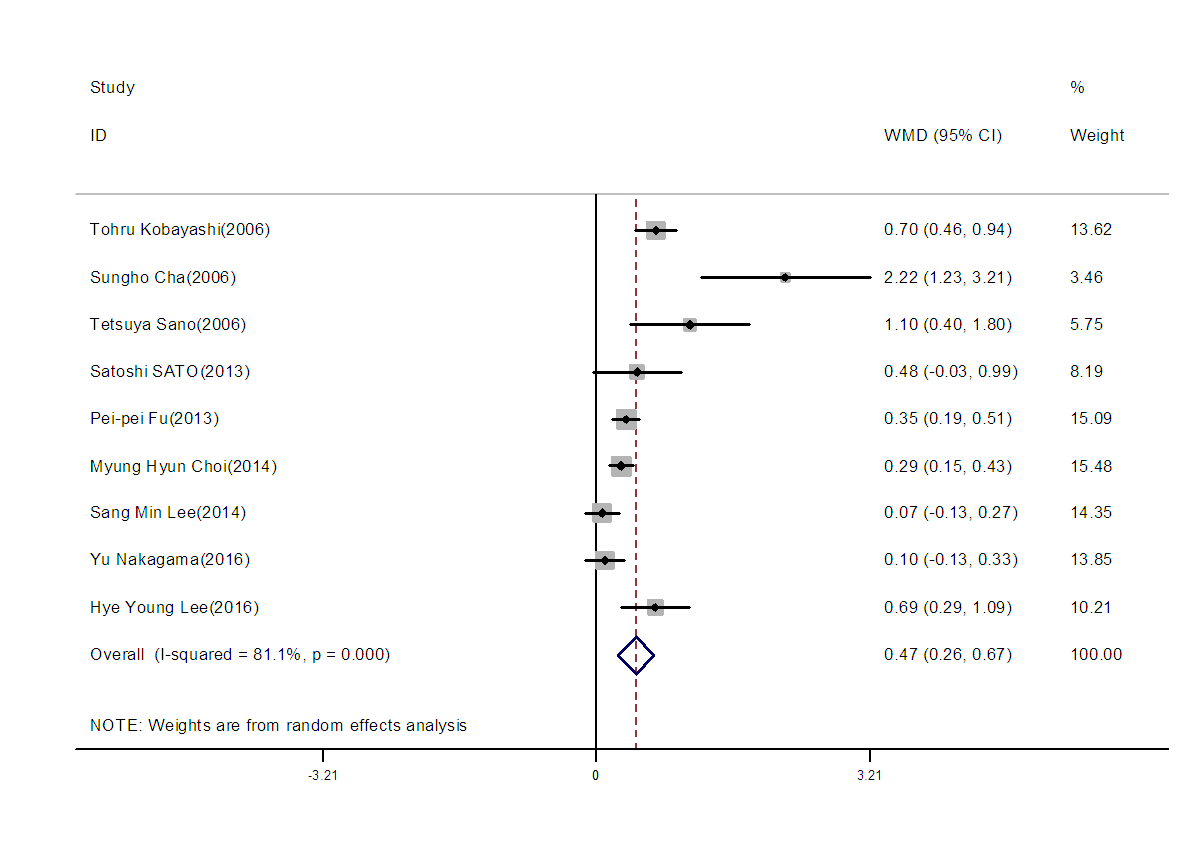
Fig. 15 Total bilirubin as a predictive index for intravenous immunoglobulin resistance in Kawasaki disease.**

**
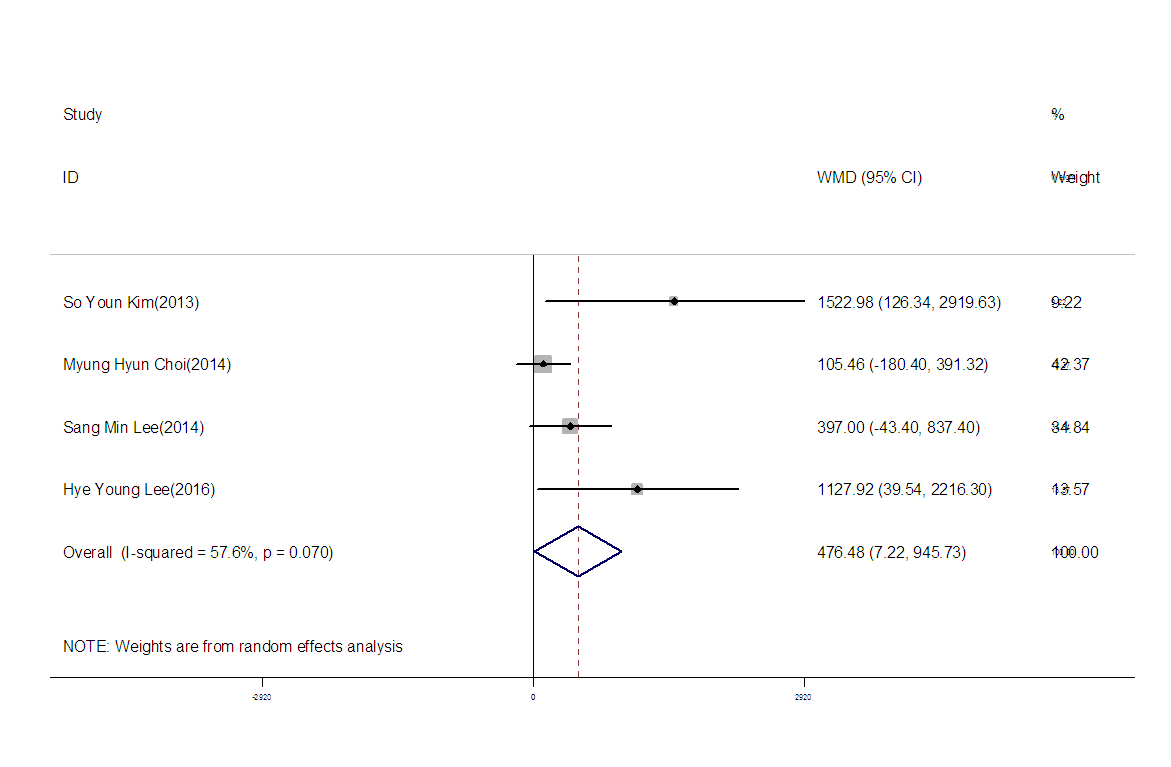
Fig. 16 Pro-BNP as a predictive index for intravenous immunoglobulin resistance in Kawasaki disease.**

**
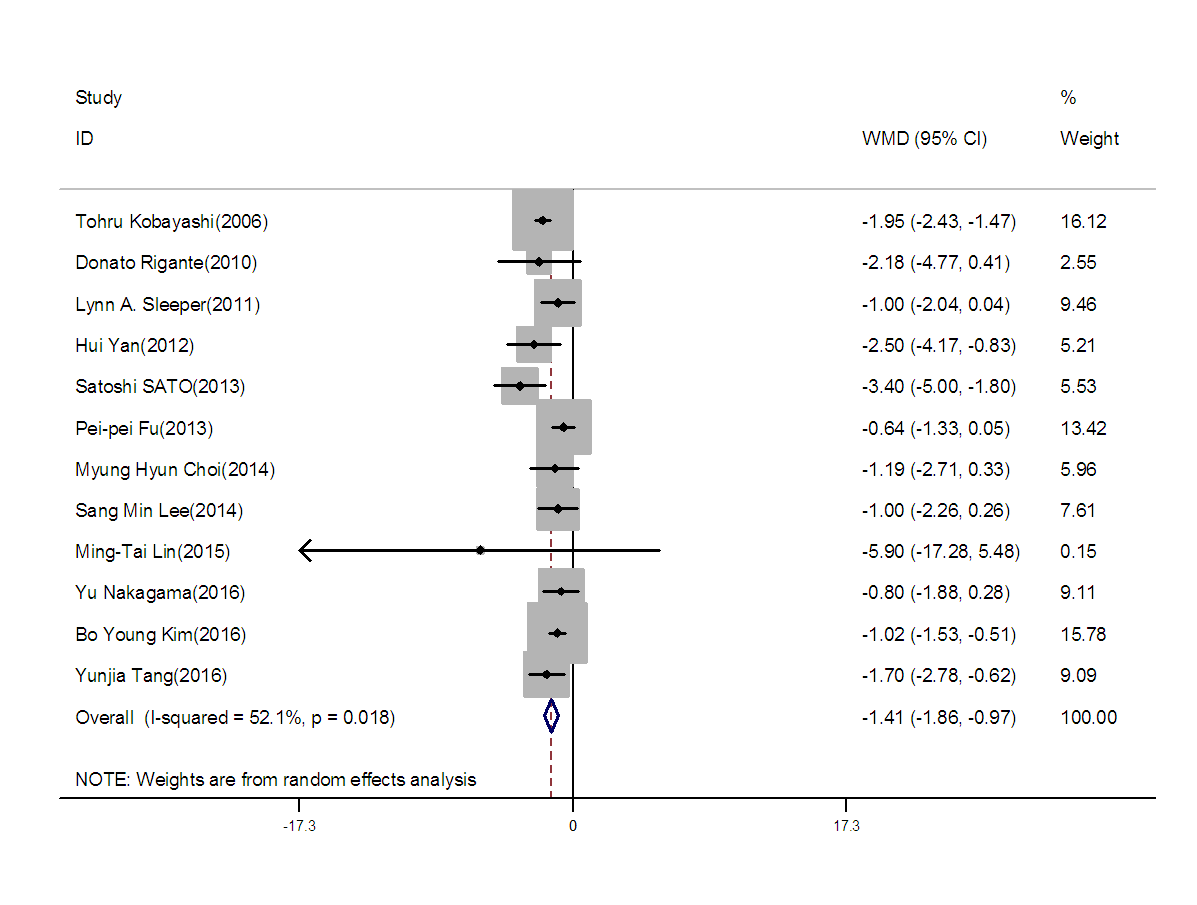
Fig. 17 Sodium as a predictive index for intravenous immunoglobulin resistance in Kawasaki disease.**

**
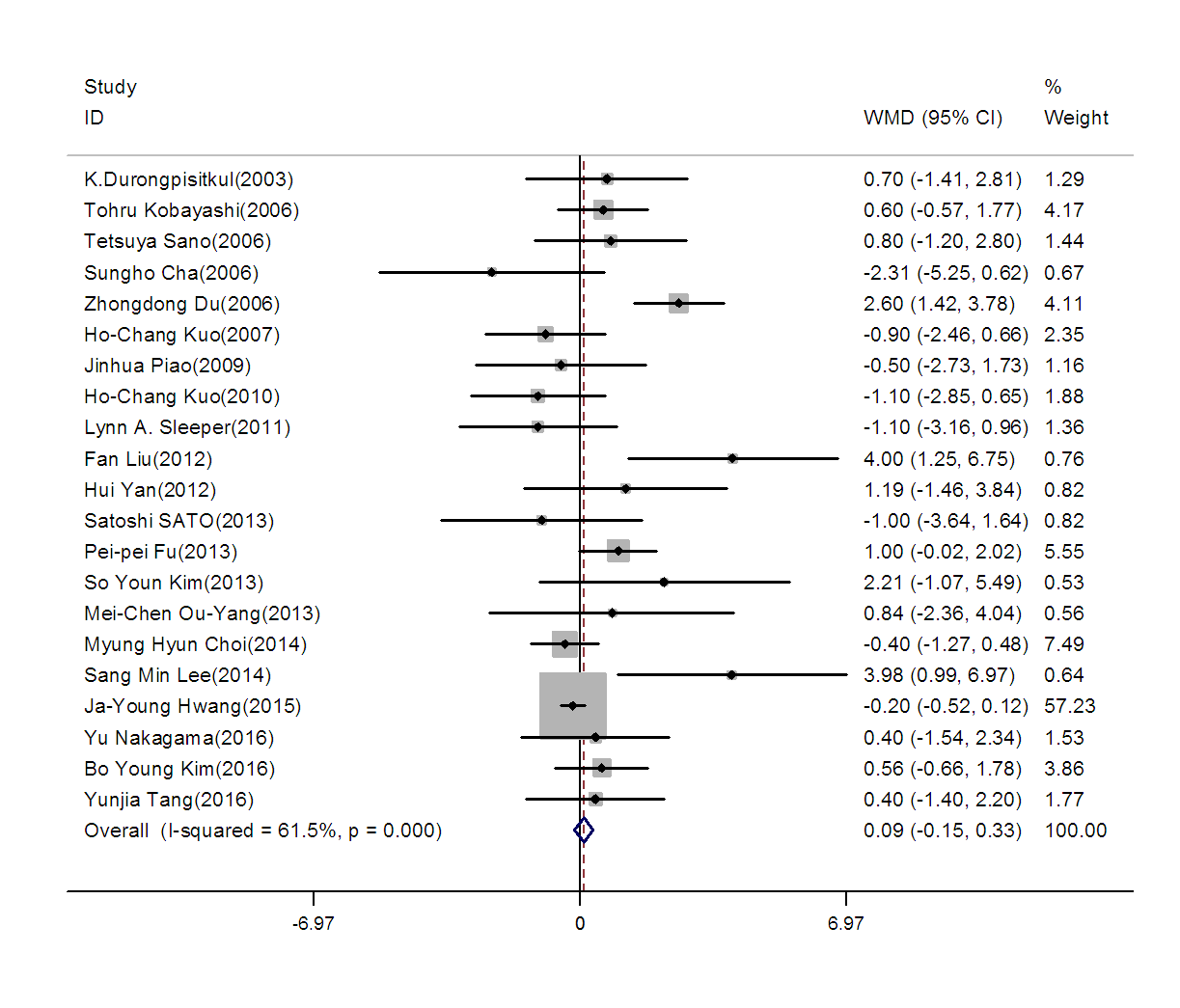
Fig. 18 White blood cells as a predictive index for intravenous immunoglobulin resistance in Kawasaki disease.**

**
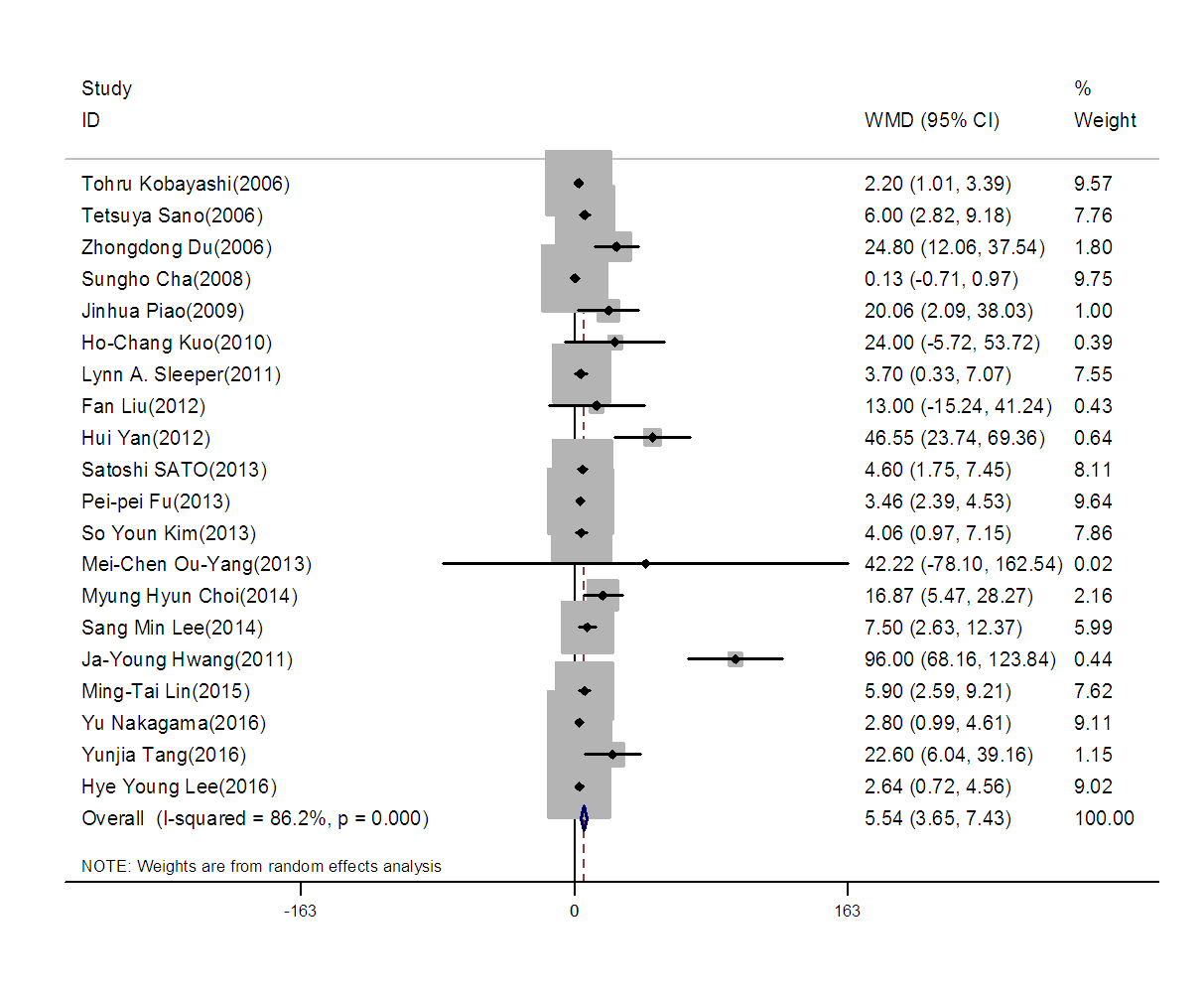
Fig. 19 CRP as a predictive index for intravenous immunoglobulin resistance in Kawasaki disease.**
